# The Effects of Sex, Age and Performance Level on Pacing in Ultra-Marathon Runners in the ‘Spartathlon’

**DOI:** 10.1186/s40798-022-00452-9

**Published:** 2022-05-13

**Authors:** Beat Knechtle, Ivan Cuk, Elias Villiger, Pantelis T. Nikolaidis, Katja Weiss, Volker Scheer, Mabliny Thuany

**Affiliations:** 1 Institute of Primary Care, University of Zurich, Zurich, Switzerland; 2 Medbase St. Gallen Am Vadianplatz, Vadianstrasse 26, 9001 St. Gallen, Switzerland; 3 Faculty of Physical Education and Sports Management, Singidunum University, Belgrade, Serbia; 4 Klinik für Allgemeine Innere Medizin, Kantonsspital St. Gallen, St. Gallen, Switzerland; 5 School of Health and Caring Sciences, University of West Attica, Athens, Greece; 6 Ultra Sports Science Foundation, 109 Boulevard de l’Europe, 69310 Pierre-Benite, France; 7 Centre of Research, Education, Innovation and Intervention in Sport (CIFI2D), Faculty of Sport, University of Porto, Porto, Portugal

**Keywords:** Ultra-marathon, Sex difference, Sport, Age, Performance

## Abstract

**Background:**

Pacing has been investigated in different kinds of ultra-marathon races, but not in one of the toughest ultra-marathons in the world, the ‘Spartathlon’.

**Objective:**

The aim of the present study was to analyse the pacing of female and male finishers competing in the ‘Spartathlon’ in regards to their age and performance groups.

**Methods:**

A total of 2598 runners (2255 men and 343 women) finishing ‘Spartathlon’ between 2011 and 2019 were analysed. We selected 10 checkpoints with split times corresponding to important race sections. Average running speed was calculated for each participant and the average checkpoint running speed for each of the 10 race checkpoints. Furthermore, to assess the pacing strategy of each runner, the percentage of change in checkpoint speed (CCS) in relation to the average race speed was calculated (for each of 10 checkpoints). Finally, the average change in checkpoint speed (ACCS) was calculated for each participant as a mean of the 10 CCSs.

**Results:**

Both women and men slowed down through the first 7 checkpoints but increased running speed towards the end of the race (reverse J-shaped pacing). Men showed a significantly greater CCS in the first and second checkpoint (*p* < 0.01 and *p* < 0.05, respectively), whereas women showed a more significant change in CCS in the last checkpoint (*p* < 0.05). Furthermore, age and sex showed no effect on ACCS, whereas ACCS differed between the performance groups. In particular, the slowest and the fastest runners showed a more minor change in ACCS than the two medium groups of both men and women (*p* < 0.01).

**Conclusions:**

In summary, successful finishers in ‘Spartathlon’ showed a reverse J-shaped pacing curve with a decrease in running speed from the start to the 7th checkpoint and an increase in running speed thereafter. This strategy was most probably due to the profile of the race course. Men showed a more significant change in checkpoint speed in the first two checkpoints, whereas women showed a more substantial change in the last checkpoint. Age and sex did not affect average checkpoint speed, whereas this speed was different between the different performance groups. The slowest and the fastest runners showed fewer changes in average checkpoint speed than the two medium groups in men and women.

## Key Points

- Successful finishers in ‘Spartathlon’ showed a reverse J-shaped pacing curve.
- Running speed decreased from the start to the 7th checkpoint and increased thereafter towards the finish.
- The slowest and the fastest runners showed minor changes in the average change in checkpoint speed than the two medium groups in both men and women.

## Background

Ultra-marathon running has gained in popularity the recent years [1–3]. As any individual sporting event (i.e., cycling, rowing, swimming) leads to peripheral fatigue [4], pacing is an essential aspect of ultra-marathon running performance. Pacing describes the way in which energy expenditure is distributed over the duration of an exercise [5] and includes different strategies such as negative pacing, all-out pacing, positive pacing, even pacing, and parabolic-shaped pacing [6]. Pacing during a race is a process of continuous decision-making, influenced by the collective behaviour of the competitors’ [7], the atmosphere around the athlete during the race [8], mood changes [9], and is associated with the goal-directed regulation of intensity [10].

Pacing has been investigated in different ultra-marathon races such as 6-h ultra-marathon running [11], 12-h ultra-marathon running [11], 24-h ultra-marathon running [11–14], in 100 miles ultra-marathon running events such as the ‘Craze Ultra-marathon [15] or the ‘Western States Endurance Run’ [16], 100-km and 161-km ultra-marathon running [17–21], 106-km trail mountain ultra-marathon [22] and the 170 km long UTMB^®^ (‘Ultra-Trail du Mont Blanc’) [23]. Most often, ultra-marathoners demonstrate a positive pacing with decreased running speeds during the race [11, 19, 22, 24].

The ‘Spartathlon’ is the oldest and one of the most demanding ultra-marathons in the world. It has been the subject of different scientific studies such as the investigation of participation and performance trends [25, 26], the sex difference in performance, the age of peak performance [26, 27], the cardiac changes following the race [28, 29], inflammatory processes [30, 31], the prevalence of exercise-associated hyponatremia [32], exertional rhabdomyolysis [33], gastrointestinal disorders [34], and bone metabolism [35]. However, no study has investigated pacing in the ‘Spartathlon’ as one of the toughest ultra-marathons in the world yet.

The ‘Spartathlon’ is a 246-km non-stop ultra-marathon where the athletes have to climb the highest point at ~ 1200 m above sea level [36], while running on tarmac roads, trails, and mountain footpaths. Pacing in ultra-marathon running in trails and hilly terrain is different from other types of running events such as road running on flat courses [37]. In particular, pacing in mountain trail ultra-marathon running is characterised by running speed variations due to the elevation changes [37, 38] and sections of positive and negative pacing [22], where the fastest times are achieved when speed fluctuations are limited [16]. For example, in the UTMB^®^ (‘Ultra-trail du Mont Blanc’), even pacing throughout the race correlated with faster overall race times [23]. An essential aspect of ultra-marathon running is that social interactions and interpersonal relationships are common in ultra-races [39].

With less variability (both negative and positive), even pacing is often the best pacing option. Namely, even pacing is considered as the most optimal pacing strategy in prolonged locomotive events, such as long-distance running, swimming, rowing, skiing, speed skating, and cycling [6]. Also, even minor speed fluctuations can result in a more significant energy cost [40].

Pacing in ultra-marathon running can also be influenced by performance level and sex [17]. In the study of Renfree et al. [17], faster 100-km ultra-marathoners started slower in race than slower competitors and increased running speed at the end of the race compared to slower runners. Women started slower in the race than men and increased running speed towards the end of the race [17].

Regarding age, pacing strategies showed no differences in age groups in the study of Renfree et al. [17]. Another study investigating 100-km age group athletes showed, however, that male age group athletes in age group 40–44 years were the best pacers showing a negative pacing in the last split of the race. For runners of all other age groups, running speed remained in the last split and decrease in runners in age group 18–24 years [41].

Considering the lack of information about pacing in the ‘Spartathlon’, and that regulation of exercise intensity is determinant for the optimal performance, especially in long-distance events, where athletes need to finish the distance in the shortest time possible, the aim of the present study was to analyse the pacing of female and male finishers competing between 2011 and 2019 in the ‘Spartathlon’ where split times were available in the results lists. Based on previous pacing findings in ultra-marathons, we expected positive pacing in the ‘Spartathlon’ where ultra-marathoners would become slower and slower during the race.

## Methods

### Ethical Approval

This study was approved by the Institutional Review Board of Kanton St. Gallen, Switzerland, with a waiver of the requirement for informed consent of the participants as the study involved the analysis of publicly available data (EKSG 01/06/2010). The study was conducted in accordance with recognized ethical standards according to the Declaration of Helsinki adopted in 1964 and revised in 2013.

### Participants

For this study, we have included official results and split times for the traditional ‘Spartathlon’ race from Athens to Sparta [36]. In total, 2598 runners from 2011 to 2019 were included in the analysis. In particular, the results of 2255 men (86.8%) and 343 women (13.2%) were analysed. Participants who did not finish the race or did not record any of the split times were excluded from the study.

### The Race

**Fig. 1:**
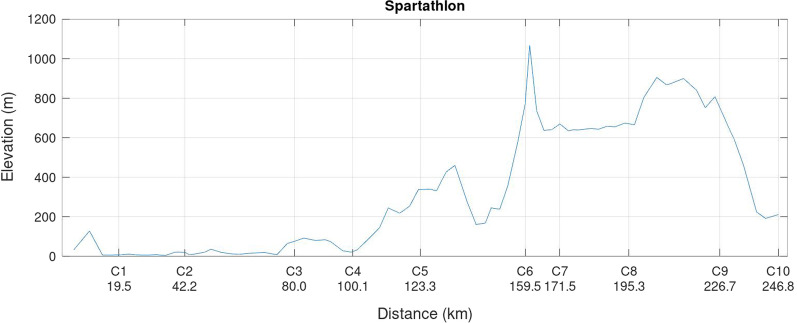
The course profile of ‘Spartathlon’

The ‘Spartathlon’ is a 246.8 km historic ultra-distance race taking place annually in September in Greece. It is one of the most challenging ultra-distance races in the world since the elevation ranges from sea level to 1200 m, is held over tarmac road, trail, and mountain footpath and must be completed within 36 h. The course profile is shown in Fig. 1. Specific entry requirements must be fulfilled to compete in ‘Spartathlon’: In the 3 years prior to the race, interested athletes must have completed an ultra-marathon such as 120 km (men) or 110 km (women) in a 12-h race, finish a 100-mile race in 21 h (men) or 22 h (women), cover 180 km (men) or 170 km (women) in a 24-h race, finish ‘Western States 100- Mile Endurance Run’ within 24 h (men) or 25 h (women), or finish ‘Badwater’ within 39 h (men) or 40 h (women) [36]. During the race, the athletes have to pass a total of 75 checkpoints within a time limit [36]. When an athlete cannot arrive at the checkpoint within the requested time limit, they are required to withdraw from the race.

In total, the ‘Spartathlon’ has 75 checkpoints, however, not all of them were used to measure split times. Therefore, we have selected 10 checkpoints with split times, corresponding to the critical race sections set by race officials [36]. In particular, the first three checkpoints in our analysis correspond to the race section from Athens to Corinth, which is 80 km long. The second race section from Corinth to Nemea is 43.3 km long, and the 4th and the 5th checkpoints were considered for this section. The third and fourth race sections (Nemea to Lyrkeia and Lyrkeia to Nestani) were combined since there are relatively short and there is no checkpoint with split time in Lyrkeia. Therefore, Nemea to Nestani is 48.2 km long with the 6th and the 7th checkpoint. Nestani to Tegea is 23.8 km long with the 8th checkpoint, and finally, Tegea to Sparta is 51.5 km long with the 9th and the 10th checkpoint (Table 1).

**Table 1:** ‘Spartathlon’ race specifications

Race sections	Checkpoint number	Checkpoint distance (km)	Official distances (km)
Athens to Corinth	1	19.5	80.0
	2	22.7	
	3	37.8	
Corinth to Nemea	4	20.1	43.3
	5	23.2	
Nemea to Nestani (via Lyrkeia)	6	36.2	48.2
	7	12.0	
Nestani to Tegea	8	23.8	23.8
Tegea to Sparta	9	31.4	51.5
	10	20.1	
Total	10	246.8	246.8

### Data Analysis

*Average race speed* was calculated for each participant and *average checkpoint speed* for each of the 10 race checkpoints (Table 1). The average race speed was calculated for each runner by dividing the race’s total distance by their final net time. Furthermore, average checkpoint speed was calculated for each runner by dividing checkpoint distances in km by the time each runner needed to cover the checkpoint distance [42, 43]. Additionally, the percentage of change in checkpoint speed (CCS) was calculated in relation to the average running speed. CCS was calculated for each of the 10 checkpoints to evaluate the pacing strategy for each runner [42]. Finally, the *average change in checkpoint speed* (ACCS) was calculated for each participant as a mean of the 10 CCSs [44]. Note that the use of both positive and negative percentage values could lower the means of CCS and ACCS. Therefore, we have transformed all percentage variables to their absolute values (i.e., only positive values were used for statistical analysis, while both positive and negative values were depicted on the graphs). Both CCS and ACCS were utilised in previous studies [42, 43]. Although speed analysis is a good choice to show pacing through the race, one dependent variable (i.e., ACCS) depicting pacing variability is often a better choice to achieve more robust results.

All conducted analyses were performed for the men and women separately. Pacing, in particular, ACCS was further evaluated by age groups and performance groups. Eight age groups were formed as follows: < 30; 30–34; 35–39; 40–44; 45–49; 50–54; 55–59; 60+ years of age [43–46]. Note that age groups with participants younger than 30 years and older than 60 years were merged since there were very few runners in these age groups (Table 2). From the entire sample, four performance groups were formed by four quartiles. Performance groups were formed based on the average running speed. They contained a high-level running group (HL < 25th percentile), a moderate to high-level running group (MHL > 25th and < 50th percentile), a moderate to low-level running group (MLL =  > 50th < 75th percentile), and a low-level running group (LL > 75th percentile) [12, 41].

**Table 2:** Participants distribution for men and women in regards to their age

Men		Women	
Years of age	*n*	Years of age	*n*
< 30	113	< 30	19
30–34	223	30–34	48
35–39	246	35–39	41
40–44	525	40–44	104
45–49	527	45–49	64
50–54	366	50–54	37
55–59	147	55–59	14
60>	84	60>	16
Missing	24	Missing	0
Total	2255	Total	343

*n* number of participants

### Statistical Analysis

Prior to all statistical tests, descriptive statistics were calculated as a mean, standard deviation, minimum and maximum values. Data distribution normality was assessed by the Kolmogorov–Smirnov (KS) test and visual inspection of histograms and QQ plots (quantile–quantile plots). Both the KS test and observed data showed a rather normal distribution. Further analysis included a mixed between-within analysis of variance (ANOVA) that was performed on CCS to test differences between checkpoints (i.e., checkpoints 1 to 10; within-subjects factor), sex (i.e., men and women; between-subjects factor) and their interaction (checkpoint × race). In addition, one two-way ANOVA was performed on ACCS to assess differences between the eight age groups (i.e., < 30; 30–34; 35–39; 40–44; 45–49; 50–54; 55–59; 60+ years of age), sex (i.e., men and women) and their interaction (age group × sex). Finally, another two-way ANOVA was also performed on ACCS to assess differences between the four performance groups (i.e., HL, MHL, MLL, and LL), sex (i.e., men and women) and their interaction (performance group × sex). For all ANOVAs, the posthoc Bonferroni test was performed. Effects size was presented via eta squared (*ŋ*^2^), where the values of 0.01, 0.06, and above 0.14 were considered small, medium, and large, respectively (Cohen). Alpha level was set at *p* ≤ 0.05. All statistical tests were performed using Microsoft Office Excel 2007 (Microsoft Corporation, Redmond, WA, USA) and SPSS 20 (IBM, Armonk, NY, USA).

## Results

**Fig. 2:**
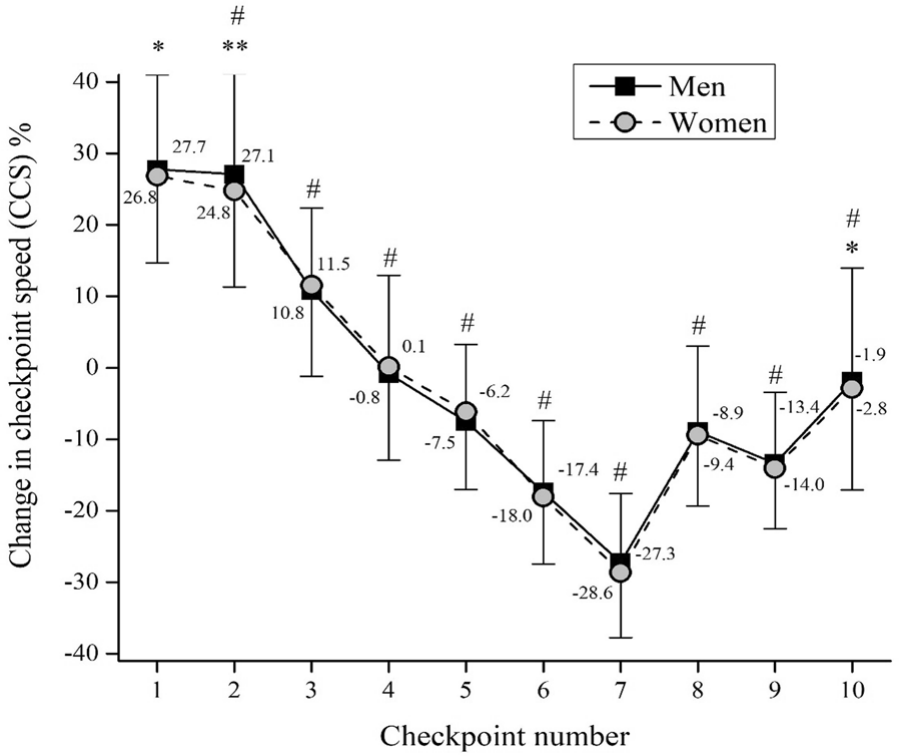
The percentage of change in checkpoint speed (CCS) in relation to the average race speed in men and women. Error bars depicts standard deviation. *Significant difference between men and women at *p* < 0.05; ** significant difference between men and women at *p* < 0.01; # significant difference between all other checkpoints *p* < 0.01

**Fig. 3:**
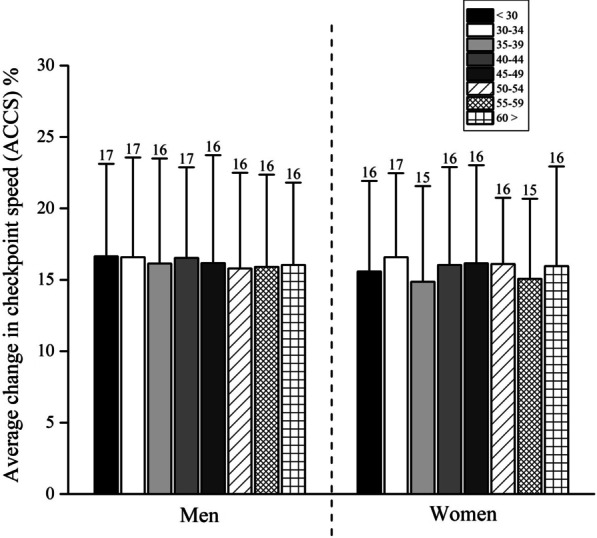
The average change in checkpoint speed (ACCS) for eight age groups for men and women

**Fig. 4:**
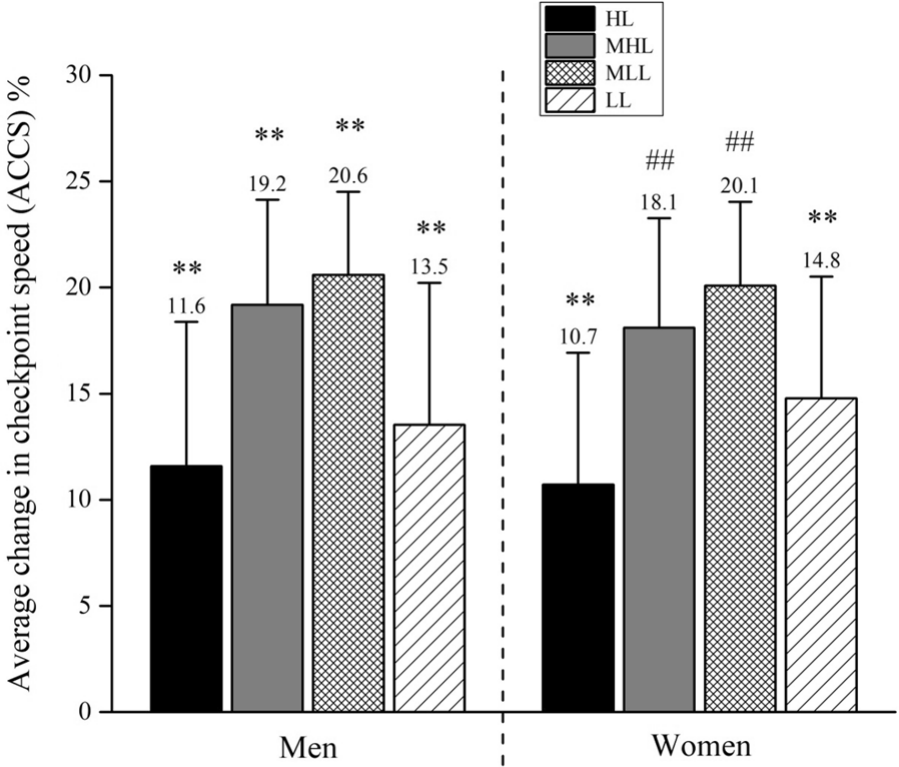
The average change in checkpoint speed (ACCS) for four performance groups for men and women. **Significant difference between all other performance groups within sex at *p* < 0.01; ## significantly different from HL and LL at *p* < 0.01. *HL* high-level, *MHL* moderate to high-level, *MLL* moderate to low-level, *LL* low-level

Participant distribution in regards to their age and performance groups are presented in Table 2**.** Average checkpoint speeds and average race speeds of participants are presented in Table 3. Regardless of their sex, ‘Spartathlon’ runners were slowing down through the first 7 checkpoints, after which they increased average running speed. Further examination of pacing is presented in Figs. 2, 3 and 4. To assess the pacing of men and women, mixed between-within ANOVA was performed on CCS (Fig. 2).

**Table 3:** Checkpoints and average race speed for men and women

Race sections	Checkpoints	Average checkpoint speed (m/s)							
		Men				Women			
		Mean	SD	Min	Max	Mean	SD	Min	Max
Athens to Corinth	Checkpoint 1	2.92	0.22	2.52	4.00	2.85	0.18	2.28	3.44
	Checkpoint 2	2.90	0.26	2.18	3.97	2.80	0.22	2.22	3.59
	Checkpoint 3	2.51	0.28	1.94	3.52	2.48	0.24	2.04	3.22
Corinth to Nemea	Checkpoint 4	2.23	0.34	1.20	3.48	2.22	0.33	1.32	4.09
	Checkpoint 5	2.07	0.31	1.42	3.13	2.07	0.29	1.60	2.93
Nemea to Nestani (via Lyrkeia)	Checkpoint 6	1.74	0.26	1.26	2.871	1.72	0.24	1.27	2.74
	Checkpoint 7	1.53	0.23	0.88	2.51	1.50	0.21	0.92	2.24
Nestani to Tegea	Checkpoint 8	1.91	0.25	1.02	3.14	1.90	0.21	1.40	2.59
Tegea to Sparta	Checkpoint 9	1.82	0.20	0.91	2.70	1.80	0.16	1.31	2.38
	Checkpoint 10	2.09	0.42	1.06	3.60	2.06	0.35	1.26	3.19
Total	Average running speed	2.22	0.32	1.18	3.40	2.21	0.28	1.41	3.12

*m/s* meters/second, *SD* standard deviation, *Min* minimumvalue, *Max* maximum value

As a result, significant main effects of checkpoint [*F*_(9,5634)_ = 1145.8, *ŋ*^2^ = 0.54, *p* < 0.01], sex [*F*_(9,5634)_ = 4.49, *ŋ*^2^ < 0.01, *p* = 0.03] and checkpoint × sex interaction [*F*_(9,5634)_ = 2.76, *ŋ*^2^ < 0.01, *p* = 0.01] were observed. Post hoc analysis showed that the CCS of each checkpoint in both men and women is significantly different than the others (*p* < 0.05). Furthermore, men showed significantly greater CCS in the first two checkpoints (*p* < 0.05; *p* < 0.01, respectively), whereas women showed significantly greater CCS (*p* < 0.05) in the last checkpoint (Fig. 2). When pacing was assessed via ACSS (Fig. 3), the two-way ANOVA showed no significant main effects of age [*F*_(15,2559)_ = 0.37, *ŋ*^2^ < 0.01, *p* = 0.92], sex [*F*_(15,2559)_ = 0.79, *ŋ*^2^ < 0.01, *p* = 0.37] and age × sex interaction [*F*_(15,2559)_ = 0.21, *ŋ*^2^ < 0.01, *p* = 0.98].

Finally, when another two-way ANOVA was applied on ACSS to further assess pacing (Fig. 4), it showed significant main effects of performance [*F*_(7,2598)_ = 162.4, *ŋ*^2^ < 0.16, *p* < 0.01] and performance × sex interaction [*F*_(7,2598)_ = 2.62, *ŋ*^2^ < 0.01, *p* = 0.05], whereas no significant main effects was found on sex [*F*_(7,2598)_ = 0.87, *ŋ*^2^ < 0.01, *p* = 0.35]. Post hoc analysis revealed that ACSS in performance groups for men were all significantly different than the others (*p* < 0.01). On the other hand, in women’s performance groups, only LL and HL were significantly different than the others (*p* < 0.01), while MHL and MLL were only different to HL and LL (*p* < 0.01) (Fig. 4).

## Discussion

The purpose of the present study was to analyse the pacing of female and male finishers competing in the ‘Spartathlon’ between 2011 and 2019. We hypothesised that successful finishers would adopt a positive pacing. The main findings were (i) both women and men slowed down through the first seven checkpoints to increase running speed towards the end of the race (reverse J-shaped pacing), (ii) men showed a more significant positive change in checkpoint running speed in the first two checkpoints, whereas women showed a significantly more significant negative change in checkpoint running speed in the last checkpoint, (iii) age and sex showed no effect on the average change in checkpoint running speed and (iv) average change in checkpoint running speed was different between the performance groups, whereas the slowest and the fastest runners showed lesser changes in checkpoint running speed than the two moderate groups.

### Reverse J-Shaped Pacing in Spartathlon

The first important finding was that both women and men slowed down at the beginning of the race (i.e., checkpoints 1 to 7) to increase running speed in the last part of the race (i.e., checkpoints 7 to 10). This overall pacing profile is called a ‘reverse J-shaped pacing’ [6] and can be explained in the ‘Spartathlon’ by the course profile. From Nemea (C6) to Nestani (C7), the runners have to climb an altitude of 960 m within 13 km to reach the Sangas Pass at ~ 1100 m above sea level.

Reverse J-shaped pacing has been reported in different ultra-marathon running situations such as 24-h ultra-marathon running [12–14], and 100 miles running in the ‘Craze Ultra-marathon’ [15]. In flat races like the 24-h ultra-marathon, the reverse J-shaped pacing might be explained by an end spurt. In 283 participants in the 24-h run in the ‘International Ultramarathon Festival’ held in Athens-Hellinikon, Greece, the runners adopted a reverse J-shaped pacing profile where faster runners were pacing more evenly and with a lower variability in the running than slower runners speed [12]. In the ‘VI Rio 24-h Marines Ultra-marathon’, the runners showed a reverse J-shaped pacing with a lower running speed at the beginning and a higher running speed towards the end [14]. Finally, in the 161-km ‘Craze Ultra-marathon, a reverse J-shaped pacing profile was found in all groups where only 38% of all finishers completed the end spurt [15]. The decrease of speed across an ultra-marathon race would be due to accumulated exercise-induced fatigue. Furthermore, the presence of an end spurt—typically observed in non-elite runners—would indicate a sub-optimal distribution of effort across the race with the runner being able to be faster in the end of the race.

The terrain is the most likely explanation for pacing in a mountain ultra-marathon. The athletes generally run slower uphill and faster in downhill sections compared to level sections [38]. For example, in a mountain ultra-marathon covering 173 km with up-and downhill sections, an increase in running speed in the last section (i.e., a speed reserve) has been found for even and uphill running [37]. Similarly, in Switzerland's ‘100-km Lauf Biel’, the fastest athletes in the age group 40–44 years increased their running speed in the last segment (i.e., negative pacing). This was most likely due to the environmental conditions with the rising sun in the dawn and the flat course in the last segment before the finish line [41]. In a 106-km trail mountain ultra-marathon, the runners combined positive pacing where speed decreased in the first 90% of the race but increased in the last 10% of the event [22]. We assume that the terrain (i.e., the course profile) was responsible for the reverse J-shaped pacing in the ‘Spartathlon’. However, there might be other factors that we have not considered, such as weather, temperature, daytime and night time running and napping/sleeping during the night. Therefore, the J-shaped pacing in the ‘Spartathlon’ should be attributed to both the course profile and typical pacing observed in ultra-marathon running races.

### The Difference in Change in Checkpoint Running Speed Between Women and Men

A second important finding was that men and women showed differences in the change in checkpoint running speed where the change was more prominent at the beginning of the race for men (checkpoints C1 and C2) but more significant at the end of the race (C10) for women. Differences in pacing have also been reported for female and male 100-km ultra-marathoners. Female competitors started slower in the race and increased running speed towards the end than male competitors [17]. A potential explanation could be that women pace differently compared to men considering their strength and physiological differences [47].

A high running speed at the start—that cannot be maintained across the race—can be due to an increased risk taking. In an experiment investigating risk perception, initial speed was associated with individual risk perception. Lower risk perception was associated with a higher running speed at the start [48]. Differences in pacing between female and male ultra-marathoners have been reported. In an analysis of 100-km ultra-marathoners, women started relatively slower than men but finished relatively faster. Faster and slower men paced differently than faster and slower women [17].

A performance difference between women and men might be explained by initial values of body weight as well as changes of body weight during the race. Or in other words, women with a lower body mass might be able to run faster in certain sections of a race, e.g., in uphill sections. In a 107-km mountain ultra-marathon, body weight changes were related to running speed changes in the first and the last section of the race. Women probably take advantage of shorter breaks at refreshing points compared to men [49]. Data covering 2,348,505 results from the six worldwide marathon races between 2009 and 2019 showed that women were 18.33% better at keeping an even pace than men. The difference in running pace between the first and the second half was 11.49% and 14.07% for women and men, respectively. Besides that, those who started more slowly were more likely to run a marathon at a more even pace than those runners that started faster [50].

### No Influence of Age and Sex on Running Speed

A third important finding was that both sex and age did not affect average checkpoint running speed. It has been reported that the sex difference in ultra-marathon running performance increases with increasing race distance and increasing finishing place, i.e. the sex difference increased with increasing race duration [51]. The sex differences also increased when fewer women and men were in an ultra-marathon [51]. In the present study, 2255 men (86.8%) and 343 women (13.2%) were analysed. The stringent selection criteria probably led to only the best women and men entering the race and finishing. A recent review assumed that women could have an advantage in ultra-endurance compared to men [47].

With regards to the role of age, no difference in pacing was observed, which was in agreement with previous studies [17, 18]. In ultra-marathon running, increasing age is not associated with a decrease in running speed [52]. In a study investigating pacing in 100-km ultra-marathon running, pacing remained consistent across age groups [17]. Also, in the ‘100 km Lauf Biel’ held in Switzerland, older athletes showed no slowing down during the race [18]. In a study investigating the age of peak ultra-marathon performance for ultra-marathoners competing in time-limited ultra-marathons held from 6 to 240 h (i.e., 10 days), the age of peak ultra-marathon performance increased and performance decreased. Furthermore, the age of peak ultra-marathon performance increased with increasing race duration and an increasing number of finishes. These athletes improved race performance with a growing number of finishes [52]. Therefore, age seems not to have a relevant influence on pacing and performance in ultra-marathon running.

Experience in ultra-marathon running is most probably the explanation for this fact [53], which is a long period of consistent training, is essential to ultra-marathoners. In summary, although the age-related decline of endurance during life-span has been well-documented, the experience and training characteristics of older ultra-marathon runners would allow them exhibiting similar pacing patterns as their younger peers.

### The Difference in the Average Change in Checkpoint Running Between Performance Groups

A last important finding was that the average change in checkpoint running speed differed between the performance groups. The average checkpoint running speed change for men differed between all four performance groups. For women, the average change in checkpoint running speed showed a difference between the high-level and the moderate-to-high level runners and a difference between the low-level runners and the moderate-to-low level runners.

This is a rather important finding and quite different from a marathon where faster runners are better pacers than younger runners [54]. Also, there were no differences between men and women (Fig. 3), only between performance groups within sex. Finally, for women, both high-level running and low-level running were different compared to both moderate-to-high level running and moderate-to-low level running athletes (Fig. 3). Also, the high-level and low-level running athletes were different. Only moderate-to-high level and moderate-to-low level running athletes were not different from each other. These results can be associated with the lowest performance variability between these groups, highlighting the few differences observed.

These findings confirm recent findings in 100-km ultra-marathon running that pacing differs for different performance levels [17, 19]. In a study investigating pacing in 100-km ultra-marathon running in the World Masters Championships, women started with a slower running speed but higher finishing speed than men. Male high-level and low-level runners paced differently, but not female runners [17]. In another study with 100-km ultra-marathon runners, differences between faster and slower runners were also described. In the ‘100 km IAU World Challenge’ held in Winschoten, Netherlands, the faster runners were running with fewer changes in running speed, started the race with a higher running speed than the slower runners and maintained their running speed for a longer distance before slowing down [19].

The difference in performance between faster and slower runners can also be explained by a ‘herd behaviour’ [13, 15]. In an analysis of pacing in 24-h ultra-marathon running, a reverse J-shaped pacing was found with a reduction in the running speed from second to last to the last hour. The fastest runners started at a relatively lower running speed and showed a more even pacing than the slower runners [13]. In a 100-miles ultra-marathon, the top runners showed a ‘herd behaviour’ by staying close together with the leading runner in the initial phase of the race [15].

### Limitations

This study is not free of limitations. Firstly, the small sample size of women (13.2%) makes generalising the information difficult. However, women generally account for ~ 10% in ultra-marathon running [47]. A further limitation is the missing of split times for earlier years and additional race checkpoints, which were, however, not available from the race results. Another limitation is that we had no data about the training and pre-race experience of these participants [53], such as details of nutrition and fluid intake would have been of interest [55] that are associated with energy distribution across the race. Furthermore, the influence of course elevation and environmental conditions such as weather was not considered [56]. Unfortunately, we could not consider physiological and pathophysiological aspects in this analysis [57]. On the other hand, the present study can offer insights for runners and coaches (especially those with few experiences in this kind of race) regarding the best training strategies and pacing during the event.

## Conclusions

In summary, successful finishers in ‘Spartathlon’ showed a reverse J-shaped pacing with a decrease in running from the start to the 7th checkpoint and an increase in running speed afterwards. This specific strategy was most probably due to the profile of the race course. Men showed a more significant change in checkpoint speed in the first two checkpoints, whereas women showed a more substantial change in the last checkpoint. Age and sex did not affect average checkpoint speed, whereas this speed was different between the different performance groups. The slowest and the fastest runners showed fewer changes in average checkpoint speed than the two medium groups in men and women. These findings may help athletes and coaches properly plan their race strategy, where runners of moderate running speed should consider pacing more even during the race.

## Data Availability

For this study, we have included official results and split times for the traditional ‘Spartathlon’ race from Athens to Sparta [36].

## Abbreviations

CCS: Change in checkpoint speed
ACCS: Average change in checkpoint speed
HL: High level
MHL: Moderate to high level
MLL: Moderate to low level
LL: Low level
QQ plots: Quantile–quantile plots
